# Investigating the relationship of government revenue and expenditure on economic growth using a generalized method of moments: Does state-level panel ensure sustainable growth?

**DOI:** 10.1371/journal.pone.0301764

**Published:** 2024-05-10

**Authors:** Sumandeep, Ravi Kiran, Rakesh Kumar Sharma

**Affiliations:** School of Humanities & Social Sciences, Thapar Institute of Engineering and Technology Deemed to be University, Patiala, India; Cavendish University / Kyambogo University, UGANDA

## Abstract

The current research project investigates the correlation between economic growth, government spending, and public revenue in seventeen Indian states spanning the years 1990 to 2020. An analysis of the relationship between key fiscal policy variables and economic growth was conducted utilising a panel data approach, the Generalised Method of Moments (GMM), and fully modified Ordinary Least Squares (FMOLS & DOLS) estimation. In our investigation, we assessed the impacts of non-tax revenue, development plan expenditure, tax revenue, and development non-plan expenditure on (i) the net state domestic product (NSDP) and (ii) the NSDP per capita. The findings indicate that the selected fiscal variables are significantly related. The results indicate that expeditious expansion of the fiscal sector is obligatory to stimulate economic growth in India and advance the actual development of the economies of these states.

## Introduction

The importance of fiscal policy in deciding the rate of growth has been emphasised in some recent literature on endogenous growth [1–3]. Strong public finance policy guarantees the economy runs smoothly. Fiscal policy dictates all decisions about taxation and spending in order to foster economic expansion. Investing in production channels by the government, using money from both direct as well as indirect taxes, boosts the economy [4, 5]. The financial strategy of the state greatly impacts the economic and social lives of the countrymen. Therefore, it is highly recommended to examine growth, fiscal performance, public tax revenue, and government spending policies.

This research endeavors to clarify the connection between tax revenue (both taxed and non-taxed), government expenditure (planned and unplanned for development), and GDP growth. Accelerating Net State Domestic Product (NSDP) and NSDP/Capita are two of the many goals that the governments of India’s individual states are working towards. There has to be an emphasis on economic growth for two reasons: (i) it’s useful to know how different parts of government spending and taxation work together to achieve this aim, and (ii) the easiest way to measure the growth of Indian states is with their NSDP and NSDP/Capita. When evaluating a state’s economic growth, the NSDP and NSDP/Capita metrics are commonly used. Thus, these are incorporated into the present study as dependent variables. The other variables that are part of the study are: Tax revenue & non-tax revenue. The governments of various states spend money on development plans and non-plans; this is known as development expenditure. This study uses panel data analysis spanning 30 years to look at how certain fiscal variables have affected the GDP growth of some Indian states.The study posits that allocating resources towards health and education significantly and positively impacts the accumulation of capital in these sectors. Consequently, this capital might stimulate economic expansion.Most studies have used a standard regression method to look at how different factors affect the variation in interregional tax revenue [6–11]. The inclusion of an error term in a regression method implies inaccuracy and, due to its stochastic nature, may result in an irrational computation of tax effort [12, 13]. employed GMM to analyse the relationship between fiscal policy and economic growth between 1999–2001 from "pooled" time-series and cross-sectional data from thirty-nine economies. The findings highlight that in order to instill greater discipline, it is preferable to direct expenditures towards subsidies, transfers, and interest payments. A prudent combination of revenue growth and cost reductions could be a more effective consolidation strategy for achieving higher returns. The research emphasised that external deficit financing isinvariably more expensive than internal borrowings.

There is no evidence to suggest that capital expenditure increases in tandem with economic growth, despite the fact that the literature indicates that capital expenditure has a greater multiplier effect on economic growth than revenue expenditure [14].Applying VAR methodology, [15] investigated the connection between the aggregate of various fiscal variables and the growth of the US. When compared across regions, these studies shed light on how taxes, spending, and GDP growth interact. Studies involving Indian states that utilized the methods of two-step GMM, FMOLS, and DOLS co-integration are, however, still lacking.

In order to enhance comprehension of the relation of government spending, taxation with economic development in seventeen selected Indian states, this study constructs an endogenous growth model. The effect of growth-promoting fiscal variables—including net state domestic product (NSDP), per capita NSDP, tax income, and government spending—has been attempted to be examined. Examining panel data from 17 selected Indian states from 1990 to 2020, the study uses fiscal variable projections, DOLS co-integration, and the Pedroni test to determine how fiscal variables will affect economic growth in these states. Amidst the aforementioned context, the study is grounded in the subsequent aims:

Q1:To compare tax structure and expenditure patterns of selected 17 Indian states based on key indicators -Tax Revenue, non-tax Revenue, developmental plan expenditure, and developmental non-plan expenditure with the NSDP and NSDP/Capita growth of respective Indian states.
Q2: To look into how the NSDP affects the spending of state governments in selected 17 Indian states.
Q3: To look into how the NSDP/Capita growth affects the spending of state governments in selected 17 Indian states.

The Indian economy is presently situated to experience both rapid and sustained growth. India’s prospects are currently engendering an unprecedented degree of optimism as a result of the convergence of several favourable variables. India’s economy, presently ranked fifth globally, continues to expand at the most rapid pace among major economies on account of strong domestic demand. Presently, India holds the position of the third-largest economy as measured by purchasing power parity (PPP). Based on the projections of the International Monetary Fund (IMF), it is expected that India’s share of global growth will increase from 16–18% by 2028.Although the Indian economy is increasingly interconnected on an international level via trade and financial networks, robust domestic demand continues to be the principal catalyst for growth. Furthermore, the potential for medium and long-term growth in the economy has been enhanced by the government’s recent structural reforms in various sectors including taxation, banking, business facilitation, manufacturing, inflation control, and digitalization. These initiatives have also placed significant emphasis on developing both physical and digital infrastructure [16].

This research aims to comprehend the significance of the Indian tax system and the changing spending trends of selected Indian states. The study’s results and findings will help to propose additional restrictions on the choice of revenue and expenditure patterns for the 17 chosen Indian state’s tax structure, considering the importance of fiscal determinants like NSDP, per capita NSDP, tax revenue, non-tax revenue, development plan expenditure, and development non-plan expenditure. Analyzing the degree to which fiscal policy influences economic growth is crucial as this would enable in identification of states that can sustain growth. An examination of the association of development expenditure, public revenue, economic growth and expansion would yield advantageous insights. This study will provide policymakers with valuable insights as it conducts a comparative analysis of the impacts of economic growth and development investment on public revenue.

It would also be beneficial to look into the relationship between public revenue, economic growth & expansion, and development expenditure. This study will be useful for policymakers because it compares and contrasts the ways in which public revenue is affected by development investment and economic growth.

Whether public revenue will increase to become a significant source of finance for public spending is critical and demands a critical analysis. Additionally, it will be examined to determine whether it appears to be a fundamental source of economic growth. This study’s first section includes an introduction and background information on the study. It describes the objectives and purpose of the investigation. Former literature is reviewed in Section 2. This review examines fiscal policy indicators and economic growth in India and globally. Section 3 describes this paper’s data and methods. Part 4 presents the study’s results. Section 5 discusses and concludes. Section 6 discusses the study’s implications and contributions.

## 2 Literature review

The relationship among capital expenditure, economic growth & tax revenue has been extensively studied. An examination of the connections between eras conducted by earlier researchers has been undertaken; however, further comprehensive research may be necessary due to the potential influence of contemporary laws and events on the findings. The challenges under investigation centre on various facets, and as the discipline progresses, an increasing number of scholars develop an interest in novel predicaments. It is imperative to thoroughly examine the viewpoints and empirical findings of additional studies prior to disseminating the results of this particular one.

### 2.1. Theoretical framework on tax, public spending and growth nexus

Most research on the "Tax-Growth" relationship examines how tax policy affects economic performance in different countries [17–22].It has always been important to research the causal relationships between tax, spending, economic growth policies, and fiscal policy. To set the scene for our analysis, it is critical to comprehend the underlying presumptions of prior research. Growth has a positive association with fiscal policy variables [23–26]. They concur with Keynesians that, in order to stimulate the economy in the short and long term, the government should fund public goods, defence, law and order, healthcare, research and development, and human capital development. Nevertheless, this perspective is not shared by all economists, as some adhere to the widely held notion that government spending hampers GDP growth ([1, 27–30].Contrary to this viewpoint, Djellout et al. (2014) [26, 31] contend that fiscal policy-related variables have minimal impact on economic expansion. Government spending on productive projects has a strong negative short-term correlation with growth, according to their findings, and it may eventually stimulate economic expansion and growth.

By employing time series data spanning from 1965–2013, [32]investigate the impact of government size on five African nations: Egypt, Ghana, Kenya, Nigeria, and South Africa. The variables examined in their analysis are openness, income risk, volatility in terms of trade, and per capita income. The results obtained from converting original data to Moving Average (MV) and applying Autoregressive Distributed Lags (ARDL) and Fully Modified OLS vary across countries due to the influence of country-specific variables. Additionally, they discovered that per capita income had a significant positive effect on the size of government in each nation. [33] re-evaluated the impact of public spending on economic growth in the Nigerian economy, specifically focusing on government fiscal expansion, capital expenditure, and recurrent expenditure. ARDL method was applied to annual time series data spanning the years 1981 to 2017 in order to conduct the impact analysis. Empirical evidence supports the assertion that there is a positive correlation between public spending metrics and economic growth in Nigeria. Furthermore, it was discovered that ongoing government spending has a major detrimental effect on economic growth. Additional findings from the Granger causality analysis indicate that the government’s fiscal expansion, which relies heavily on debt financing, has a significant Granger causal effect on domestic investment and public spending.

In their 2021 study, [34] examine the relation that exists between corruption and economic activity. Corruption was assessed using the corruption perception index (CPI), and data collection from 2012 to 2019 was conducted by the researchers through a panel comprising 48 industrialized and developing nations. The findings demonstrate that corruption has an adverse effect on the economy, particularly on the growth and magnitude of GDP per capita. However, in comparison to small governments, the benefits of corruption reduction are comparatively limited for large governments. [35, 36] have identified an inverted U-shaped correlation between growth rate and public expenditure. [37] examined the correlation between government spending and economic growth in South Asian countries (SANs), the BRICS, and other emerging economies from 2007 to 2016 using the inverted U-shaped hypothesis of the Armey curve. GMM and system GMM panel modelling were utilised to conduct threshold-old regression. It was found that public expenditures significantly stimulated economic growth; however, they could only offer transient resolutions to challenges within a country.

### 2.2. Fiscal policy and dynamics of macroeconomic indicators in the Indian context

Analysts of Indian policy are intrigued by the evaluation of tax initiatives and their future prospects. A considerable number of academics have endeavored to assess and quantify India’s efficacy in tax revenue generation, with a particular focus on the subnational [38–41]. The tax endeavours and potential of Indian states were verified by Karnik and Raju (2015) [42] through the utilisation of stochastic frontier analysis (SFA). Several scholars have examined the variation in taxes among Indian states using regression analysis [10, 38, 43–46].However, literature suggests that the empirical relation between tax structure and growth performance in India remains ambiguous and unclear. This research endeavours to address the existing knowledge gap by examining the effects of tax revenue, non-tax revenue, Development plan expenditure, and Development Non-Plan Expenditure on (i) net state domestic product (NSDP) and (ii) NSDP/Capita.

According to research conducted in Asia and Africa, a higher multiplier effect is responsible for the observed increase in per-capital income when the percentage of capital investment rises [47, 48]. In general, deficits hinder economic growth by undermining public trust in the government. Using panel data analysis from 30 countries, a negative relationship has been established [49, 50]. used co-integration analysis to find that GDP would fall by 0.21% if the budget deficit were to rise by 1%.

[51] conducted a study on the management of public finances in the North-west states, of India. Financial records from 1980 to 2010 have been examined for four states in North-west India, viz. Punjab, Haryana, Himachal Pradesh, and Jammu & Kashmir. The results indicate that states need to improve their performance to become financially self-sufficient. Using budgetary indicators, [52] performed an analysis at the state level. The results reflected that the surplus in the revenue account is useful for building infrastructure. [53] studied the tax policies of seventeen states in India to find ways to free up government funds. Not only is the total state own-tax revenue looked at, but four separate state-level taxes are as well. These are state sales tax, state excise tax, state stamp duty and registration fees, and state motor vehicle taxes. They developed the SFA models to determine the degree of inefficiency in tax collection from 2000–2001 to 2010–2011. According to the results, technological inefficiencies are the main reason states aren’t able to collect as much money as they could. Reasons for the drop-in tax efforts in 1991–92 and 2010–11 were investigated by [54]. They argued that increased inter-governmental transfers reduce states’ tax efforts, while increased political competitiveness, law and order, and public spending as a percentage of GDP increase states’ tax efforts. An improvement in the administrative setup might assist in enhancing tax efficiency, according to [41] tax efficiency index, which showed a considerable degree of variation across states. From 2001 to 2014, he used SFA to evaluate the tax efforts of 16 important states. In 2023, [55] used the stochastic frontier panel data model to evaluate the taxability and tax effort of seventeen of India’s most populous states from 2001 to 2017. The study found that the states’ ability to levy taxes has been diminished due to the Goods and Services Tax.

This study investigatesthe relationship between key fiscal policy variables and economic growth. It examines the effects of tax revenue, non-tax revenue, development plan expenditure, and development non-plan expenditure on (i) net state domestic product (NSDP) and (ii) NSDP/Capita.This research attempts contribute to the existing literature: (i) by providing fresh research and addressing a need by examining the impact of fiscal policy on economic growth in a selection of 17 Indian states, as well as numerous studies conducted prior to the structural modifications of the GST and Demonetization reform that combined data from earlier and later periods.(ii) Effort is also made to analyze the connection of fiscal variables with economic growth of selected 17 Indian states.

The relationship between fiscal variables and government spending is crucial in identifying the optimal model to enhance economic growth, spending efficiency, and government revenues.This study will thus help the Indian government implement policies for economic growth. This research takes a holistic approach by developing two models that interchange these endogenous variables. This study uses panel data, the GMM, and FMOLS along with DOLS estimation to predict all variables and provide a greater depth into the association between fiscal policy variables and growth. Previous studies mostly used economic growth as an endogenous variable.Limited studies have created multiple models using interchangeable variables. NSDP and NSDP/capita are endogenous variables in the two models, while tax revenues, non-tax revenues, developmental plan expenditures, and developmental non-plan expenditures are exogenous. Predicting economic growth in 17 Indian states using exogenous variables may not be enough. However, predicting variables is crucial.

## 3. Research design and methodology

### 3.1. Theoretical framework of the method

Growth is positively correlated with fiscal policy variables according to several studies [9, 49, 56–59]. Some argue that Government spending limits GDP growth [39, 60, 61]. According to [62]the relationship between government revenue and expenditure in a group of seventeen emerging South Asian countries, utilising GMM. [63] analysed the relationship between government revenue and expenditure in G-7 countries using the frequency domain approach of econometrics. Further Jain et al. (2021) [37] also used GMM and system GMM panel modelling to examine government spending and economic growth in SANs, the BRICS, and other emerging economies from 2007 to 2016. They found that public expenditures stimulated economic growth but could only solve short-term problems. Based on above studies, the current research creates an endogenous growth model using GMM and FMOLS to improve understanding of the connection of government spending and taxation with economic development in seventeen chosen Indian states. DOLS co-integration, and the Pedroni test have been applied to assess the impact of fiscal variables on economic growth.

### 3.2 Type and sources of data

An analysis has been conducted on the impact of fiscal variables that promote growth, such as net state domestic product (NSDP), per capita NSDP, tax income, and government spending. The study analyses panel data from 17 chosen Indian states between 1990 and 2020. It utilises fiscal variable projections. The present study drew upon secondary data from the RBI publication, State Finances.The study has included 17 states of India viz. Andhra Pradesh, Bihar, Chhattisgarh, Goa, Gujarat, Haryana, Jharkhand, Karnataka, Kerala, Madhya Pradesh, Maharashtra, Orissa, Punjab, Rajasthan, Tamil Nadu, Uttar Pradesh and, West Bengal.

### 3.3. Model specification

The models used in this study use a variety of methods to predict the economic growth of the individual states in India. The first technique is panel unit-root analysis. Furthermore, the results from each technique were verified using the generalized method of moment (GMM) and fully modified OLS (FMOLS), DOLS co-integration.Pedroni and unconstrained Co-integration Rank tests (Trace Value and maximum Eigen value) were used to empirically examine the relationship between public revenue, government spending, and economic growth in selected 17 states from 1990 to 2020, assuming the error term is serially uncorrelated.

### 3.4 Estimation technique and justification of system GMM

GMM method allows incorporation of a specific advantage of dynamic estimators over static estimators and help control the endogeneity of the lagged dependent variable in a dynamic model, predominantly when a correlation is identified between the explanatory variables and the error term. GMM also mitigates the effects of unobserved panel heterogeneity and omitted variable bias. First differences transformation (FD) is frequently employed in the empirical literature concerning dynamic models (GMM). This practice can be partially attributed to the findings of Arellano and Bond (1991) [64]. Subsequently, Arellano and Bover (1995) [65] proposed forward orthogonal deviations (FOD) as a substitute for the first difference transformation. Various configurations of the GMM model (FOD) panel are utilised in this study.This study usesdifferent configurations for the GMM model (FOD) panel.According to [66], among the estimators for instrumental variables, GMM is the most effective. This study employed the two-step GMM estimation method. For large-scale, reasonably-long-term data sets where disturbances are anticipated to display heteroscedasticity, two-step GMM outperforms one-step estimate, according to a number of prior studies [64, 67].Specification of the dynamic model for NSDP is:

$$\text{Y}\;\text{it}={\alpha \text{Y}}_{\text{i},\text{t}-\text{1}}+\text{Xit}\;\beta +{\text{e}}_{\text{t}}+{\text{u}}_{\text{it}}$$

This can be defined as:

Where α: scalar, β: vector of coefficients (kx1).
In this basic structure, Yit: NSDP) and X´it: vector of explanatoryvariables.

Similar to various panel economic growth models as highlighted by [68] in debate of Mankiw and Islam, we incorporate non-tax revenues, developmental plan expenditures, and developmental non-plan expenditures in addition to fiscal variables, in order to evaluate the impact on economic activity and growth. Thesubscript (i) indicates the chosen 17 states across time-period (t). The terms u_it_and e_t_represent a composite error, where the random component of the variation in ourindependent variable is derived from the idiosyncratic error (u_it_) and the time-invarianterror, e_t_.Lagged dependent variable Y_it-1_ is introducedas a determinant for the dynamic panel. In the basic configuration, we assume that the maximum sample period (t) isequal to 30 years with 17 selected Indian states (i). In order to estimate our models using the GMM method, we employ orthogonal deviations as a transformation option to eliminate the specification’s influence. Furthermore, the GMM specification conforms to the Arellano-Bond two-step process.

[69] suggests a completely revamped (FMOLS) estimator for panels with diverse and co integrated variables. This approach tackles simultaneity bias as well as non-stationary regressor issues. Pedroniexpands on the work of [70] by applying panel data analysis to the semi-parametric correction to OLS estimator, which was considered as a means of removing the second-order bias caused by the regressors’ endogeneity. After the dependent variable is corrected for endogeneity using the long-run covariance matrices, the variables are then estimated using the simple-OLS method by FMOLS estimator.

To confirm the significance of the results from previous studies and determine whether the results of the methodologies are consistent, we consider other panel data estimation techniques. It is important to ensure that the dependent and predictor variable data series are stationary at the level before applying FMOLS & DOLS. In addition, co-integration in the model is the second necessary condition to be considered which is supposed to be developed using OLS. To find out if a data series is stationary, three separate unit root tests were employed: [71–73]. These tests were performed with constant and with constant and trend (C & T), without constant and without trend (none), and with constant and trend. When performing the unconstrained Johansen co-integration test, the maximum Eigenvalue and trace value conditions were considered. FMOLS & and co-integration tests (FMOLS, DOLS) were used to find out if there is a relationship between several explanatory variables. Using the firm-specific determinants, two regression equations are created to generate the models, and E-views 10 application software is used to regress the data. The GMM methodology was used to confirm the results of the earlier techniques.

Equations for the different models

1. NSDP = β1 (Tax Revenue) + β2 (Non-tax revenue) + β3 (Development plan expenditure) + β4 (Development non-plan expenditure) +ε_it_
2. NSDP/Capita = β1 (Tax Revenue) + β2 (Non-tax revenue) + β3(Development plan expenditure) + β4(Development non-plan expenditure) +ε_it_

In these equations, β1–β4 represents the slope of each external variable, and ε_it_ for the error term.

## 4. Results and discussion

The dependent variables NSDP and NSDP/Capita are shown in Table 1 along with their descriptive statistics. Spending on the Development Plan, Taxes, Other Sources of Revenue, and Development spending are predictors. The standard deviation of different independent variables reflects that all these variables are volatile for the period (2000–2020). According to Jarque and Bera’s (1987) [74] test, all three variables’ data series obey normality [75]. Additionally, the skewness must ±1 to satisfy the normalcy requirements. In a similar vein, Kurtosis’ value ought to be less than 3 for normal distribution. Some researchers believe it to be ±2. [76, 77].

**Table 1:** Descriptive statistics of variables.

	NSDP	NSDP/Capita	Tax Revenue	Non-Tax Revenue	Development plan expenditure	Development Non-Plan Expenditure
Mean	13.964	10.499	11.811	10.451	10.986	11.797
Median	14.089	10.473	11.913	10.497	10.897	11.859
Maximum	16.428	12.457	14.256	13.416	13.301	13.881
Minimum	9.840	8.846	8.129	7.878	7.363	8.686
Std. Dev	1.014	0.663	1.135	0.815	1.135	0.925
Skewness	-0.831	0.291	-0.652	-0.221	-0.484	-0.556
Kurtosis	4.658	2.927	3.463	3.066	3.519	3.768
Jarque-Bera	112.341	7.029	39.033	4.075	24.660	37.243
Probability	4.031	0.029**	3.341	0.130	4.415	8.180
Sum	6828.757	5134.389	5775.909	5110.849	5372.200	5768.748
Sum Sq. Dev.	502.551	214.826	629.285	324.371	628.688	418.245
Observations	489	489	489	489	489	489

Source: Author’s Calculations with E-views. [Note: **significant at 5%]

### 4.1 Descriptive statistics

From Table 1, it can be concluded that the data for some variables do not meet these two normality conditions. All the variables, NSDP, Tax Revenue, non-tax Revenue, Development plan expenditure, and Development Non-Plan Expenditure, have the normality of data series (p-value>0.05); which indicates acceptance of the alternate hypothesis that data is normal. Standard deviation of the variables highlights that the data series of the two dependent variables are far more stable.

Table 2 displays the correlation matrix of all six variables. NSDP has high correlation with all exogenous variables, viz. non-tax revenue; tax revenue and development expenditure. In contrast, the NSDP/Capita has low correlation with all the exogenous variables. Development plan expenditure shows a high degree of association with Development Non-Plan Expenditure (0.893).This is followed by tax revenue (0.845) and NSDP (0.817). Development Non-Plan Expenditures are highly correlated with NSDP, an endogenous variable (0.920), tax revenue, and development plan expenditure. There is a strong correlation between tax revenue and the growth of non-plan expenditures, but non-tax revenue alone does not prove this. Lastly, NSDP and other exogenous variables are strongly correlated with tax revenue.

**Table 2:** Correlation matrix of variables.

	NSDP	Per Capita NSDP	Development plan expenditure	Development Non-Plan Expenditure	Non-Tax Revenue	Tax Revenue
NSDP	1	0.235	0.817	0.920	0.665	0.877
Per Capita NSDP	0.235	1	0.203	0.191	0.352	0.326
Development plan expenditure	0.817	0.203	1	0.893	0.653	0.845
Development Non-Plan Expenditure	0.920	0.191	0.893	1	0.709	0.901
Non-Tax Revenue	0.665	0.352	0.653	0.709	1	0.758
Tax Revenue	0.877	0.326	0.845	0.901	0.758	1

Source: Author’s Calculations with E-views.

Table 3 displays the statistics of unit roots using different methods. The first step was to conduct unit root tests at three distinct levels: without intercept and trend, with intercept, and with both intercept and trend.

**Table 3:** Unit root at first difference.

	NSDP	Per Capita NSDP	Tax Revenue	Non-Tax Revenue	Developmental Plan Expenditure	Developmental Non-Plan Expenditure
LLC						
None	-14.299 (0.000)***	-11.353 (0.000)***	-7.145 (0.000)***	-14.580 (0.000)***	-11.464 (0.000)***	-11.427 (0.000)***
With C	-15.117 (0.000)***	-11.392 (0.000)***	-6.053 (0.000)***	-6.594 (0.000)***	-6.850 (0.000)***	-7.726 (0.000)***
With C & T	-12.717 (0.000)***	-8.876 (0.000)***	-3.050 (0.001)***	-4.0347 (0.000)***	-5.639 (0.000)***	-4.366 (0.000)***
Breitung T-stat						
With C						
With C & T	-10.933 (0.000)***	-11.589 (0.000)***	-4.701 (0.000)***	-6.243 (0.000)***	-6.273 (0.000)***	-6.469 (0.000)***
Im, Pesaran and Shin W-stat						
None						
With C	-13.461 (0.000)***	-10.457 (0.000)***	-10.805 (0.000)***	-11.991 (0.000)***	-9.843 (0.000)***	-12.107 (0.000)***
With C & T	-11.391 (0.0155)**	-7.742 (0.000)***	-9.023 (0.000)***	-10.0187 (0.000)***	-8.025 (0.000)***	-10.460 (0.000)***
ADF—Fisher Chi-square						
None	231.815 (0.000)***	172.649 (0.000)***	107.946 (0.000)***	261.245 (0.000)***	180.822 (0.000)***	186.199 (0.000)***
With C	227.400 (0.000)***	170.815 (0.000)***	177.003 (0.000)***	200.646 (0.000)***	159.025 (0.000)***	202.095 (0.000)***
With C & T	177.916 (0.000)***	119.922 (0.000)***	138.101 (0.000)***	157.351 (0.000)***	123.068 (0.000)***	163.801 (0.000)***
PP						
None	382.245 (0.000)***	346.444 (0.000)***	213.500 (0.000)***	541.223 (0.000)***	389.753 (0.000)***	412.947 (0.000)***
With C	350.116 (0.000)***	358.489 (0.000)***	399.520 (0.000)***	394.431 (0.000)***	400.194 (0.000)***	407.659 (0.000)***
With C & T	292.836 (0.000)***	343.680 (0.000)***	273.008 (0.000)***	1035.88 (0.000)***	336.284 (0.000)***	1321.03 (0.000)***

Source: Author’s Calculations with E-views [Note: ***significant at 1%; ** 5%; 10%]

### 4.2 Unit-root tests

All of the unit root tests had p-values below 0.001, which means that we can accept the hypothesis that the data series is stationary. The first difference determines the stationary state of all independent variables according to LLC. This includes tax and non-tax revenue, development plan expense, and development non-planned expense. All variables are stationary according to the Breitung T-statistic, which is used when the p-values for both the independent and dependent variables are less than 0.001. All four variables are shown to be stationarity using Shin W-Stat and Im Pearson. All variables, including tax revenue, non-tax revenue, development plan expenditure, and development non-planned expenditure, are shown to be stationary with constant intents based on the results of the ADF Fisher Chi-square test and the Phillip-Perron (PP) test. The unit root tests show that all the variables, including two internal ones, are stationary at first difference, including the exogenous ones.

### 4.3 Cointegration test

Pedroni provides seven distinct tests to determine if co-integration is present. For the unit root tests on the estimated residuals, pooling the autoregressive coefficients across different panel members forms the basis of the first four tests, which are based on pooling along the "within" dimension.

The last three tests use pooling along the "between" dimension, or averaging the autoregressive coefficients for each panelist, to conduct the unit root tests on the computed residuals. The outcomes of each of the seven Pedroni tests are presented in Table 4. The null hypothesis of "no co integration" is rejected when the p-value for both models is consistently less than 0.05 or less than 0.01 at three distinct levels (i.e., without C and T, with C, and with T), evidence of co-integration between two models is presented.

**Table 4:** Pedroni residual co-integration tests.

Tests	NSDP	Per Capita NSDP
Panel V-Statistic		
Without C and T	-2.427 (0.992)	-0.663 (0.000)***
With C but no T	-3.643 (0.999)	-1.969 (0.975)
With C and T	-5.655 (1.000)	-4.106 (1.000)
Panel rho-Statistic		
Without C and T	-5.974 (0.000)***	-5.133 (0.000)***
With C but no T	-5.313 (0.000)***	-3.891 (0.000)***
With C and T	-3.376 (0.000)***	-1.767 (0.385)
Panel PP-Statistic		
Without C and T	-12.352 (0.000)***	-12.658 (0.000)***
With C but no T	-14.266 (0.000)***	-12.952 (0.000)***
With C and T	-14.775 (0.000)***	-14.285 (0.000)***
Panel ADF-Statistic		
Without C and T	-6.187 (0.000)***	-6.536 (0.000)***
With C but no T	-7.593 (0.000)***	-6.049(0.000)***
With C and T	-7.182 (0.000)***	-6.157 (0.000)***
Group rho-Statistic		
Without C and T	-4.710 (0.000)***	-4.564 (0.000)***
With C but no T	-3.618 (0.000)***	-2.881 (0.0020)**
With C and T	-1.445 (0.0741)*	-0.525 (0.299)
Group PP-Statistic		
Without C and T	-14.716 (0.000)***	-17.324 (0.000)***
With C but no T	-16.055 (0.000)***	-17.683 (0.000)***
With C and T	-15.950 (0.000)***	-27.088 (0.000)***
Group ADF-Statistic		
Without C and T	-6.495 (0.000)***	-7.361 (0.000)***
With C but no T	-7.791 (0.000)***	-6.141 (0.000)***
With C and T	-6.735 (0.000)***	-6.297 (0.000)***

Source: Author’s Calculations with E-views.[Note: ***significant at 1%; ** 5%; 10%]

Tables 5 and 6 the findings of the Johansen co-integration test using Fisher criteria were undertaken. In the first model of NSDP, in all 17 equations, the trace values are relatively higher, and the p-values are less than 0.05. Similarly using maximum Eigen value criteria, the p-values are less than 0.05 for all 17 equations, which means the existence of co-integration in the first model of NSDP. Fisher’s test of co-integration is also carried out for model 2-related NSDP per capita. The findings of this test are also similar to the previous model. Again, the trace values are quite higher and the p-values are less than 0.05 for all 17 states. According to the maximum eigen value, the associated p-values are less than 0.05 for the majority of states. Hence, it can be concluded that in both the models, there is co-integration and we can proceed to use co-integration regression for examining the impact of different exogenous variables on NSDP and per capita NSDP.

**Table 5:** Model 1 (NSDP) unrestricted co-integration rank test (trace value & maximum eigen value).

Hypothesized	Trace Test	Prob.	Max-Eigen		At Most 1 0.05		Max-Eigen 0.05	
No. of CE(s)	Trace Test	Prob.	Statistic	Prob.	Trace Test	Prob.	Statistic	Prob.
At most 1 *	109.661	0.000***	42.414	0.003***	67.246	0.0003***	31.865	0.0132
At most 2 *	102.098	0.000***	38.921	0.011***	63.177	0.001***	25.693	0.085*
At most 3 *	104.116	0.000***	55.644	0.000***	48.472	0.0437**	27.882	0.045**
At most 4 *	86.276	0.001***	39.116	0.010***	47.159	0.050**	22.0305	0.218
At most 5 *	102.807	0.000***	38.301	0.013**	64.505	0.0007***	26.464	0.069*
At most 6 *	120.807	0.000***	54.361	0.000***	65.660	0.0005***	29.810	0.025**
At most 7 *	152.141	0.000***	101.522	0.000***	50.619	0.026**	24.807	0.108
At most 8 *	92.291	0.0003**	37.906	0.015**	54.384	0.010***	19.190	0.399
At most 9 *	120.403	0.000***	45.601	0.001***	74.801	0.000***	31.390	0.015**
At most 10 *	91.020	0.0004***	31.636	0.090**	59.384	0.0029**	21.203	0.264
At most 11 *	98.348	0.0001***	34.266	0.044**	64.082	0.0008**	30.524	0.020**
At most 12 *	106.978	0.000***	53.066	0.000***	53.912	0.0121**	21.254	0.261
At most 13 *	131.966	0.000***	57.144	0.000***	74.822	0.000***	30.254	0.022**
At most 14 *	123.098	0.001***	43.577	0.002***	79.525	0.000***	35.634	0.003**
At most 15 *	96.922	0.0001***	30.048	0.134	66.874	0.0003***	22.542	0.193
At most 16 *	114.183	0.000***	48.871	0.000***	65.311	0.0005**	35.016	0.0046**
At most 17 *	96.639	0.0001***	38.070	0.014**	58.568	0.0036**	26.310	0.0721*

Source: Author’s Calculations with E-views.[Note: ***significant at 1%; ** 5%; 10%]

**Table 6:** Model 1 (Per Capita NSDP) unrestricted cointegration rank test (trace value & maximum eigenvalue).

Hypothesized	Trace Test	Prob.	Max-Eigen		At Most 1 0.05		Max-Eigen 0.05	
No. of CE(s)	Trace Test	Prob.	Statistic	Prob.	Trace Test	Prob.	Statistic	Prob.
At most 1 *	118.019	0.000***	43.688	0.002***	74.331	0.000***	34.318	0.005**
At most 2 *	88.627	0.0008***	33.812	0.050**	54.815	0.009**	25.987	0.078*
At most 3 *	92.182	0.0003***	36.245	0.025**	55.937	0.007**	28.291	0.040**
At most 4 *	76.711	0.0127**	30.826	0.110	45.885	0.075*	21.921	0.040**
At most 5 *	80.857	0.0050**	29.404	0.155	51.452	0.022**	26.623	0.066*
At most 6 *	88.451	0.0008***	35.406	0.032**	53.044	0.015**	28.269	0.040**
At most 7 *	124.452	0.000***	81.367	0.000***	43.085	0.130	28.629	0.036**
At most 8 *	91.632	0.0004***	44.393	0.002**	47.238	0.050 **	21.169	0.0266**
At most 9 *	104.342	0.000***	43.299	0.002**	61.043	0.001***	33.127	0.008**
At most 10 *	92.785	0.0003***	32.570	0.071*	60.214	0.002**	28.892	0.033**
At most 11 *	94.910	0.0002***	38.637	0.012**	56.273	0.006**	25.504	0.090*
At most 12 *	86.674	0.0013**	39.670	0.009**	47.004	0.060*	18.459	0.0457**
At most 13 *	119.296	0.000***	65.596	0.000***	53.699	0.012**	28.371	0.039**
At most 14 *	87.147	0.0011**	35.779	0.029**	51.368	0.022**	24.585	0.0115**
At most 15 *	75.525	0.0163**	27.944	0.216	47.580	0.050*	20.998	0.0276**
At most 16 *	86.808	0.0012**	45.319	0.001***	41.488	0.173	21.649	0.0238**
At most 17 *	91.544	0.0004***	36.028	0.0273**	55.516	0.008**	24.837	0.0100*

Source: Author’s Calculations with E-views.

### 4.4 Estimation of GMM model

GMM estimates instrumental variables best, according to [66]. This research used two GMM estimation steps. One-step estimation is less efficient than two-step GMM for disturbances expected to exhibit heteroscedasticity in large sample data over a long time period, according to several studies. The lagged dependent variable, valid predictor variables, and no autocorrelation in the model’s error term are needed for GMM estimation. Three tests by [64] were suggested. Try to rule out first-order serial correlation and autocorrelation in the model’s error term. No serial correlation null hypothesis yields a standard normal test statistic. Under the null hypothesis of no serial correlation, the error term of the GMM model, which is normally distributed, ought not to exhibit second-order autocorrelation. [78] J-statistics test for over-identifying limitations is third. This asymptotically distributes as Chi-square under instrument validity null and judges.

Results of the GMM estimation for the two alternative models are presented in Tables 7 and 8 along with other explanatory variables including tax revenue, non-tax revenue, development plan expenditure, and development non-planned expenditure. In Model-1, all explanatory factors, viz. non-tax revenue; development plan, and non-plan expenditure are also found to be significant (p≤.05) for NSDP.

**Table 7:** Panel two stepsGMM using NSDP as a dependent variable.

	Coefficients	Standard Error	T-Stat	P-value
Lag Values of NSDP	-0.1453	0.003	-39.332	0.000***
Tax Revenue	0.090	0.100	0.900	0.3814
Non-Tax Revenue	0.036	0.011	3.274	0.0048**
Developmental Plan Expenditure	-0.097	0.025	-3.784	0.0016**
Developmental Non-Plan Expenditure	-0.147	0.045	-3.227	0.0050*
Cross-Section Fixed Effects First Difference				
Mean Dependent variable		0.0029		
Sum Squared residuals		50.484		
J- Statistic		15.754 (0.202)		
Standard Error of regression		0.341		
S.D. Dependent Variable		0.359		
Observations		438		
Dependent Variable = NSDP				

Source: Author’s Calculations with E-views. [Note: ***significant at 1%; ** 5%; 10%]

**Table 8:** Panel two-step GMM using NSDP/Capita as dependent variable.

	Co-efficients	Std. Error	T-Stat	Prob.
Lag Values of Per Capita NSDP	0.770	0.060	12.784	0.0000***
Tax Revenue	0.099	0.036	2.694	0.0159**
Non-Tax Revenue	-0.0076	0.023	-0.321	0.751
Developmental Plan Expenditure	0.0141	0.020	0.703	0.491
Developmental Non-Plan Expenditure	0.060	0.021	2.820	0.012**
Cross-Section Fixed Effects at First Difference				
Mean Dependent variable		0.050		
Sum Squared residual		10.718		
J- Statistic		15.983 (0.250)		
Standard Error of regression		0.154		
S.D. Dependent Variable		0.107		
Observations		438		
Dependent Variable = Per Capita NSDP				

Source: Author’s Calculations with E-views.[Note: ***significant at 1%; ** 5%; 10%]

For the second model, the NSDP/Capita, tax revenue, and development non-plan expenditure are significant (p≤.05). In both the models, the explanatory variables had a positive relation with NSDP and NSDP/Capita. For both the models, the Sargan—Hansen test or Sargan’s J values are 15.754 and 15.983 respectively. The Sargan—Hansen test results for both the models demonstrate the validity of the instruments and the lack of association between error terms and instruments. As the p-value of J-stat exceeds 0.05, this signifies that the instruments are valid, authentic and un- correlated with the latter.

**Fig 1:**
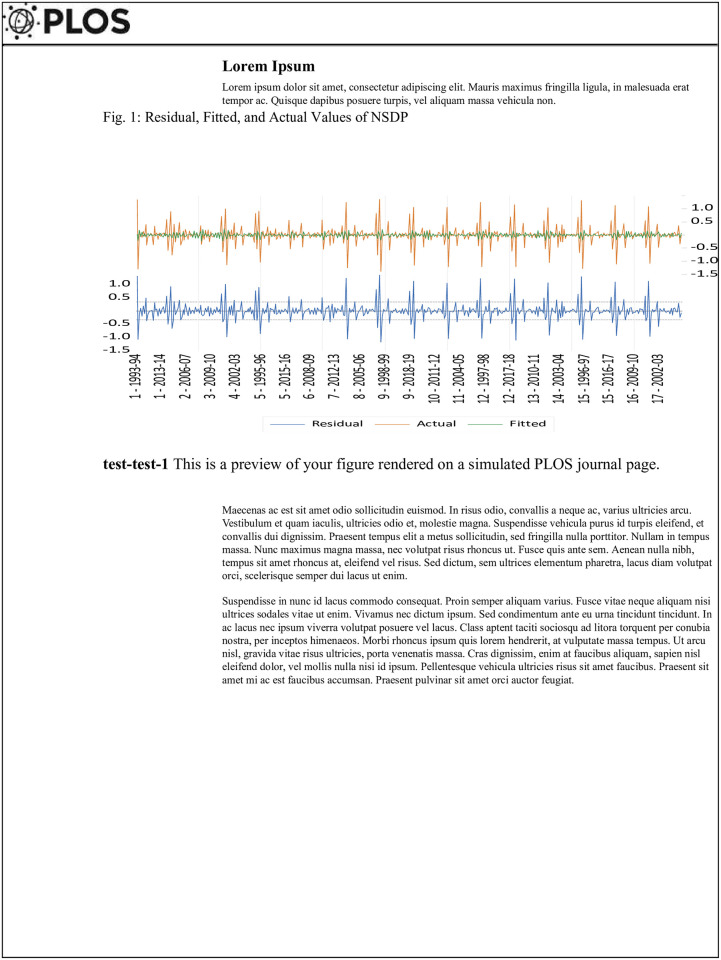
Residual, fitted, and actual values of NSDP. It is graphical residual representations along with actual and fitted values NSDP &NSDP/Capita using the GMM model. As we can see the residual or error terms of the model presented through blue color are within the limit of -1 to +1. While actual and fitted values of NSDP are moving alongside. There is not much deviation between the actual and fitted values of NSDP. Table 8: Panel two-step GMM using NSDP/Capita as dependent variable.

**Fig 2:**
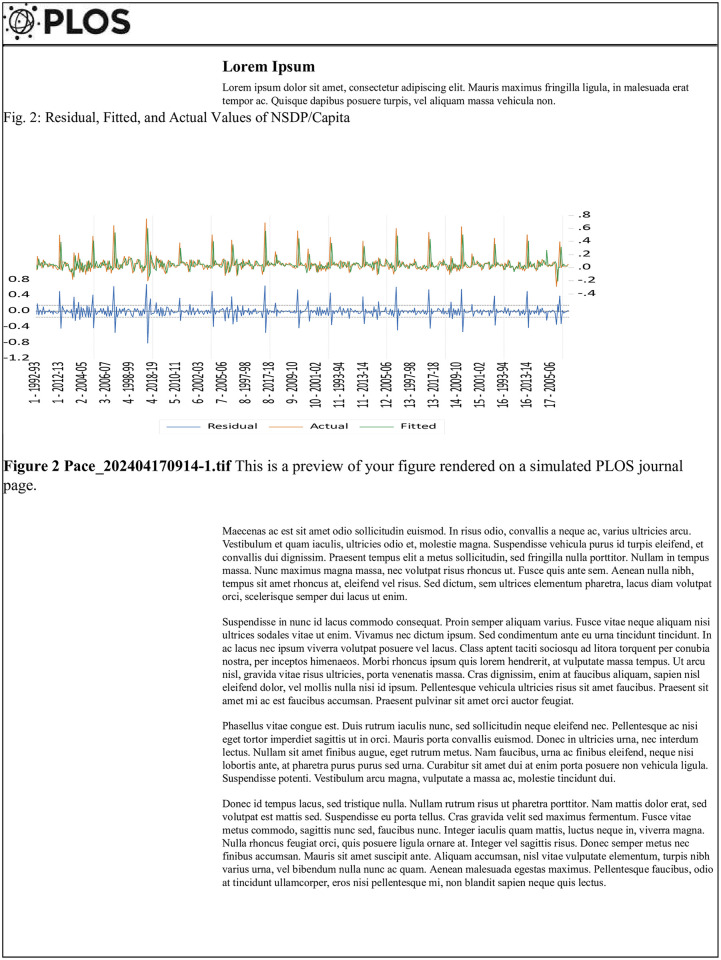
Residual, fitted, and actual values of NSDP/capita. It is demonstrates graphically the residual, fitted, and actual values of per capita NSDP. As we can see the residual or error terms of the model presented through blue color are within the limit of -0.4 to +0.4. While actual and fitted values of NSDP per capita are moving alongside. There is not much deviation between the actual and fitted values of NSDP. Considering the residual, actual, and fitted values above two GMM models can be considered as good fit models and these further can be used to forecast NSDP and per capita NSDP.

The serial correlation between NSDP and NSDP/Capita, as determined for the first order in model two (NSDP/capita), is deemed statistically significant at the 10% level (p<0.10). Applying the methodology devised by [64], it is observed that both models exhibit a negative serial correlation. As a result of the correlation of the second order, the null hypothesis is accepted. The statistical analysis reveals that the first model exhibits a significant first-order serial correlation at the 1% level, while the second model does not demonstrate such significance. In model two, neither the first-order nor the second-order serial correlations exhibit statistical significance [64] (Figs 1 and 2).

The OLS estimator for heterogeneous panel data is extensively modified and proposed by Pedroni (2000) and fully updated OLS estimation approach to the endogeneity-corrected variables was then applied.

### 4.5 Estimation of FMOLS models

Table 9 provides fully adjusted OLS estimates of co-integrated connections for the two alternative model specifications, NSDP and NSDP/Capita. In Model-1 (NSDP), the fiscal measures, tax revenue, and development non-plan expenditure is affirmative and significant. The relation is inverse for Non-Tax Revenue and Development Plan Expenditure with NSDP and is also not significant. Conversely, in Model-2 (NSDP/Capita), all other variables (tax revenue, expenditure on the developmental plan, and development non-plan expenditure) are positive and they are significant, however the relation is negative and insignificant for non-tax revenue.

**Table 9:** Fully modified ordinary least square (FMOLS) estimation model-1 (NSDP) and Model-2 (Per Capita NSDP).

S	FMOLS		FMOLS	
Explanatory variables	Model-1	T Statistics	Model-2	T Statisticsi
	NSDP		NSDP/Capita	
Sample	1489		1489	
NSDP	--	--		
Per Capita NSDP	--	--		
Tax Revenue	0.247	3.810 (0.0002)*	0.083	2.082 (0.0378)**
Non-Tax Revenue	-0.0002	-0.004 (0.996)	-0.004	-0.146 (0.883)***
Development Plan Expenditure	-0.0394	-0.628 (0.529)	0.126	3.271 (0.0012)*
Development Non-Plan Expenditure	0.683	7.045 (0.000)*	0.471	7.878 (0.000)*
R Square	0.898		0.930	
Sum Squared Residuals	47.217		14.340	
Adj. R Square	0.894		0.927	
S.E. of Regression	0.323		0.178	
	
Long-Run Variance	0.186		0.070	
Mean Dependent Variable	14.008		10.523	
S.D. Dependent Variable	0.994		0.660	

Source: Author’s Calculations with E-views. [Note: ***significant at 1%; ** 5%; 10%]

Based on 30-year period growth rates were calculated and ranks were provided to all states for selected variables (Tables 10 and 11). In terms of NSDP and NSDP/Capita the states that can sustain growth are Maharashtra, Gujarat Tamil Nadu, and Andhra Pradesh. Goa has to improve in terms of Revenue and Expenditure. Uttar Pradesh Has a lower rank in NSDP/Capita, but has rank 2 in tax revenue collection and Development non- Planned Expenditure and is at first rank in terms of Development Planned Expenditure. Punjab though at 2 ranks in NSDP is not performing well in terms of revenue and expenditure. Haryana needs to improve in terms of tax revenue and planned and non-planned expenditure. Bihar and Odisha indicate a lackluster performance. Rajasthan is also among the poor performers.

**Table 10:** Growth rates of states for selected indicators.

State	NSDP Growth Rate	NSDP/Capita Growth Rate	Tax Revenue (1990–2020)	Non-Tax Revenue (1990–2020)	Development Planned Expenditure (1990–2020)	Development Non-Planned Expenditure (1990–2020)
Andhra Pradesh	5.93	4.20	4.98	4.59	4.53	5.00
Bihar	5.56	3.86	4.5	4.18	4.22	4.79
Chhattisgarh	6.39	4.17	4.46	4.05	4.53	4.36
Goa	4.47	4.58	3.6	3.67	3.08	3.79
Gujarat	5.78	4.34	4.93	4.48	4.29	4.97
Haryana	5.65	4.41	4.55	4.44	3.92	4.53
Jharkhand	5.48	4.17	4.5	4.16	4.48	4.51
Karnataka	5.71	4.23	4.9	4.33	4.45	4.79
Kerala	5.51	4.25	4.72	3.76	4.22	4.64
Madhya Pradesh	5.72	4.15	4.75	4.47	4.46	4.82
Maharashtra	6.16	4.43	5.24	4.79	4.55	5.19
Odessa	5.36	4.05	4.25	3.86	4.21	4.42
Punjab	5.62	4.48	4.66	4.48	3.9	4.68
Rajasthan	5.66	4.17	4.63	4.33	4.2	4.75
Tamil Nadu	5.85	4.29	5.04	4.21	4.45	5.02
Uttar Pradesh	6.05	4.09	5.22	4.36	4.61	5.07
West Bengal	5.92	4.26	3.79	3.88	4.28	4.87

Source: Author’s Calculations.

**Table 11:** Rank of states based on growth rates for selected indicators.

State	Rank NSDP	Rank NSDP/Capita	Rank Tax Revenue	Rank Non-Tax Revenue	Rank (Development Planned Expenditure)	Rank (Development Non-Planned Expenditure)
Andhra Pradesh	4	10	4	2	3.5	4
Bihar	13	17	12	11	12	8.5
Chhattisgarh	1	12	14	13	3.5	16
Goa	17	1	17	17	17	17
Gujarat	7	5	5	3.5	9	5
Haryana	11	4	11	6	15	13
Jharkhand	15	12	12	12	5	14
Karnataka	9	9	6	8	7	8.5
Kerala	14	8	8	16	11	12
Madhya Pradesh	8	14	7	5	6	6
Maharashtra	2	3	1	1	2	1
Odisha	16	16	15	15	13	15
Punjab	12	2	9	3.5	16	11
Rajasthan	10	12	10	8	14	10
Tamil Nadu	6	6	3	10	7	3
Uttar Pradesh	3	15	2	7	1	2
West Bengal	5	7	16	14	10	6

Source: Author’s Calculations.

## 5.Conclusion

This panel unit root test examines how tax revenue, non-tax revenue, development plan expenditure, and development non-plan expenditure affect economic growth in Indian states. FMOLS and DOLS, a two-step GMM, were applied to 17 Indian states’ 1990–2020 annual data. This research seeks to conceptualise the relationship between government spending and taxation and economic expansion ups and downs.With the intention of offering a conceptual structure for considering the impact of government spending and taxation on the dynamics of economic growth in both the short and long term.The Keynesians and Wagner’s Law both argue that more government spending will lead to more economic growth. As Wagner’s Law (1883) states, "the higher the output, the higher the spending."[79] The significance of government expenditure in fostering economic development is indisputable. Considerable research has been devoted to examining the practical implications of Wagner’s Law [1, 80–85].In other words, an increase in the proportion of national income allocated to public expenditure results in a decline in both national income and national per capita income, as postulated by Keynesians. Irrespective of the particulars, investment has consistently stimulated expansion throughout history. The Indian context offered no substantiating evidence for Wagner’s Law. In contrast, the relationship between India’s GDP and government expenditure can be described as Keynesian.Evidenceby [32] suggest a level relationship between public spending indicators and Nigerian economic growth. Recurrent government expenditures negatively impacted economic growth over the study period, while public capital expenditures did not.

Significant results were obtained for the alternative measures of NSDP and NSDP/Capita in both the alternative models’ GMM estimations. The Johansen co-integration test following Fisher criteria shows that Models 1 (NSDP) and 2 (Per Capita NSDP) are co-integrated. We next use co-integration regression to find out how various exogenous variables affect NSDP and NSDP per capita, since both models contain co-integration. The most important results fiscal measures, tax revenue, and development non-plan expenditure are positive and significant in Model-1 (NSDP) indicating that NSDP levels rise in tandem with tax revenue levels. [52]added credence to the idea that the surplus in the revenue account is good for building infrastructure.In Model-2 (NSDP/Capita), tax revenue, developmental plan expenditure, and development non-plan expenditure are positive and significant, but non-tax revenue is negative and insignificant.The growth-based rankings given to states further support these findings; for example, Andhra Pradesh, Gujarat, and Maharashtra all rank highly for revenue and expenditure, and NSDP and NSDP/Capita are all high. A positive relation has been observed between development expenditure and tax revenue. Consistent with Keynesian theory, [86] also showed that total government expenditure positively affected GDP growth. Consequently, in order to spur economic expansion and quicken GDP growth, the government should enhance development spending on capital projects. Overspending by the government was associated with better economic growth in the long run [49, 87]. showed that states with discretionary spending, like Gujarat and Karnataka, spend less than 4% of GDP, while states like Himachal Pradesh spend 8 to 12%. This highlights the degree of state variation. This is still apparent today. To encourage investment in states that are falling behind, the study suggests certain policies to increase development expenditure on capital projects.However, caution is needed as indicated by [37]where the researchers evidenced that increased public spending before or after the optimal threshold level leads to a significant increase or decrease in growth rate, suggesting a non-monotonic relationship. Public spending thus may only be a short-term solution to crises in any nation, according to the study.Moreover, public spending decisions must be well weighed upon before implementing them.

## 6. Implications of the study

According to the GMM model, NSDP is positively and significantly related with tax revenue and development non-plan expenditure as highlighted through model 1. Model-2 provides evidence of a positive and statistically significant correlation between NSDP/Capita and development non-plan expenditure, tax revenue, and developmental plan expenditure. In light of this, it appears that states should prioritize spending and tax income on the development plan in order to raise NSDP and NSDP/Capita. Consequently, in order to accelerate NSDP and NSDP/Capita, the state governments should boost development expenditure on capital projects. The positive relation between inflated government spending and long-term economic growth was pointed out by [87]. According to the study’s findings, tax administration should prioritize raising tax revenue in order to speed up economic progress. An increase in development spending is also necessary to build better infrastructure, which would boost economic production. Strict budgetary regulations ought to be dismantled. The growth of NSDP and NSDP/Capita is supported by empirical evidence of fiscal indicator growth rates in Andhra Pradesh, Gujarat, and Maharashtra.

## Supporting information

S1 Dataset (XLSX)
